# Special Computed Tomography Imaging Features of Thymic Cyst

**DOI:** 10.1155/2022/6837774

**Published:** 2022-10-11

**Authors:** Zhi-Liang He, Zhao-Yang Wang, Zhi-Ying Ji

**Affiliations:** ^1^Minimally Invasive Intervention Center, Dezhou People's Hospital, Dezhou 253000, China; ^2^Department of Radiology, Dezhou People's Hospital, Dezhou 253000, China

## Abstract

**Objective:**

To explore the features and diagnostic value of computed tomography (CT) imaging in cases of thymic cysts.

**Methods:**

A total of 24 cases of the thymic cysts (confirmed by postoperative pathology) were retrospectively analyzed. The location, morphology, and density of the thymic cysts were summarized, and the changes in CT value of the region of interest (ROI) in the thymic cysts between noncontrast enhanced and enhanced chest scans were compared and classified.

**Results:**

The average long-axis dimension was 17.50 ± 6.00 mm, the CT value range across the 24 cases was 5–81 HU, and the average CT value of the noncontrast enhanced scans was 39.75 ± 20.66 HU. The CT value in the noncontrast enhanced scan was >20 HU in 79% of the sample cases. The CT value in the ROI of the thymic cysts under enhanced scan showed a significant decrease in 15 cases, a significant increase in 5 cases, and an insignificant change in 4 cases.

**Conclusion:**

The CT values of the thymic cysts in the enhanced scans were generally lower than in the noncontrast enhanced scans, which might be a valuable finding for thymic cysts diagnosis.

## 1. Introduction

Thymic cysts are considered to be a relatively rare type of anterior mediastinal mass; they are reported to account for approximately 1%–3% of cases [1]. With the growing popularity of low-dose chest computed tomography (CT) screening, the detection rate of thymic cysts is also increasing [2]. The density of thymic cysts is often not the typical liquid density, and the CT diagnosis rate is not high, leading to thymic cysts often being misdiagnosed as thymomas or other types of solid mass and being removed by resection [3–6]. In the present study, the CT imaging data from 24 patients with thymic cysts (confirmed by operation in the researchers' hospital) were retrospectively investigated, and the imaging changes between the noncontrast enhanced and enhanced scans were summarized and analyzed. The aim of the study was to improve the accuracy of CT-based diagnosis of thymic cysts.

## 2. Materials and Methods

### 2.1. General Characteristics

A total of 24 patients with a preoperative imaging diagnosis of thymoma and confirmed thymic cysts by thoracoscopic surgery between February 2013 and October 2019 were enrolled. The sample included 10 males and 14 females, with a male-to-female ratio of 1 : 1.4. The subjects' age range was 36–79 years, and the average age was 55 ± 11 years. Among these patients, 16 cases showed no obvious clinical symptoms and were detected by routine physical examination; 7 cases were seen for symptoms such as cough and chest pain, and 1 case was diagnosed as an accidental finding of esophageal cancer due to eating obstruction. None of the 24 patients had myasthenia gravis during their time at the hospital.

### 2.2. Apparatus and Parameters

The scan apparatuses utilized were the GE Optima CT660 128-slice CT and the United Imaging UCT 760 128-slice CT. The scan position was supine, and the scanned area ranged from the entrance of the thorax to the lower edge of the diaphragm. The detailed parameters for CT acquisition were as follows: tube voltage, 120 kVp; and tube current, 120–250 mAs with automatic exposure control. The original scanning slice thickness is 1.0 mm–1.25 mm. The slice thickness and the slice spacing in the image reconstruction were both set at 5 mm. In all 24 cases, a noncontrast enhanced scan was performed first, and then a dual-phase enhanced scan of the aortic phase (20 s) and delayed phase (40–45 s) was performed by injecting the contrast medium through the median cubital vein. The contrast agent was 80 mL of iohexol, and the injection rate was 2.5–3.5 ml/s.

### 2.3. Image Analysis

All the images were read by three thoracic radiologists and/or senior attending physicians, and a consensus was reached through consultation in cases of disagreement. The evaluated measures were as follows: (1) cyst location, morphology, and density; and (2) changes in CT value. Compared with the value in the noncontrast enhanced scan, changes of >10 HU in the CT value at the same level and location of the region of interest (ROI) in the enhanced scan were regarded as representing an increase or decrease in the CT value [7]. The evaluation standards were as follows. (1) The same level of thymic cysts in different scan phases was selected as the ROI. If the morphology changed between each phase, then the largest axial interface of the thymic cysts in different phases was selected. The area of ROI selected by each examiner and the morphology of each phase were as large as possible. The area remained unchanged to ensure an effective comparison of the CT values in each phase. (2) In the two phases of the enhanced scan, if the CT value of the ROI at the same level and location in any phase was lower than that of the noncontrast enhanced scan by > 10 HU, it was classed as a decreased CT value. (3) If there was no decrease in the CT value in the two phases, and the CT value of the ROI in any phase was higher than that in the noncontrast enhanced scan by > 10 HU, the CT value was considered to be increased. (4) During the triple-phase scan, if the CT value changed at the same level and the same location did not exceed 10 HU, the case was listed as showing an unobvious change in CT value.

## 3. Results

### 3.1. Location, Morphology, and Density

**Figure 1:**
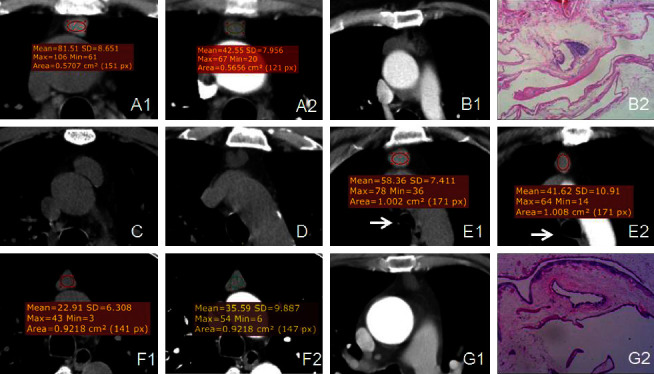
CT findings and histopathological characteristics (a): Male patient, 65 years old, with thymic cyst: (A1) CT value of 81 HU in the noncontrast enhanced scan; (A2) CT value of 42 HU at the arterial phase. (b): Female patient, 60 years old, with a rough left margin of the thymic cysts. The pathological results detected a thymic cysts with type-A thymoma (HE 4 × 10). (c): Male patient, 40 years old, with calcification of the inferior wall of the thymic cysts. (d): Male patient, 56 years old, with marginal lobulation on the edge of the cyst in the CT scan. (e): Female patient, 57 years old; thymic cysts detected by physical examination. E1 = exhalation state and E2 = inhalation state (white arrow). There were changes in the CT value and morphology of the cyst in different phases. (f): Male patient, 53 years old, with thymic cysts. Enhanced scan suggested mild enhancement of the cyst. (g): Female patient, 65 years old. The CT showed no obvious structure of the cyst wall. The pathological results suggested a multilocular cystic thymic cysts (HE 10 × 10).

The thymic cysts in the present study were all located in the anterior mediastinum. The average long-axis dimension measurement was 17.50 ± 6.00 mm. All 24 cases had uniform density. The average noncontrast enhanced scan CT value was 39.75 ± 20.66 HU, and the lowest and highest noncontrast enhanced scan CT values were approximately 5 and 81 HU, respectively (Figure 1(a)). Among the sample, 19/24 cases (79%) had a noncontrast enhanced scan CT value of  > 20 HU. Two cases had rough edges, one of which was small and surrounded by normal thymus with rough edges, and one had a rough cyst wall on the left (Figure 1(b)). Dotted calcifications on the cyst wall were visible in one case (Figure 1(c)), shallow lobular changes at the edge were found in one case (Figure 1(d)), and the remaining 20 cases had smooth cyst walls. Among the 24 cases, the cyst shapes observed were as follows: triangular/chestnut-shaped (12/24, 50%), round shapes (7/24, 29%), and flat or irregular shapes (5/24, 21%). Three cases showed significant changes in the triple-phase scan (Figure 1(e)). The location, morphology, and density of the cysts are illustrated in Table 1.

### 3.2. Changes of Computed Tomography Value in the Enhanced Scan

In the present study, a total of 15 cases (63%) had a lower CT value in the enhanced scan than in the noncontrast enhanced scan. Among these cases, 12 showed a 10–20 HU reduction, 2 showed a 20–30 HU reduction, 1 showed a 30–40 HU reduction, and the maximum decrease was 40 HU (Figure 1(a)). A total of 5 cases (21%) displayed a higher CT value in the enhanced scan than in the noncontrast enhanced scan, and the increases were in the range of 12–22 HU (Figure 1(f)). There were 4 cases (16%) in which the change in CT value between the enhanced scan and the noncontrast enhanced scan was not obvious (Table 1).

### 3.3. Histopathology

The intraoperative and gross specimens of the 24 cases showed that the cysts were soft in texture and that there existed normal thymus tissue on the cyst walls. The flat, cubic, or columnar epithelium and some cilia were visible on the cyst walls. The pathology in one case demonstrated a multilocular thymic cysts (Figure 1(g)), and the structure of the multilocular cyst wall was not visible on the enhanced CT scan. Among the observed cases, 23 were congenital thymic cysts, of which one case was combined with type-A thymoma on the cyst wall (Figure 1(b)) with limited marginal roughness in the CT scan. The remaining cyst walls were smooth.

## 4. Discussion

Thymic cysts are categorized as congenital thymic cysts or acquired (secondary) thymic cysts according to their origin. Congenital thymic cysts are mainly caused by unclosed thymic pharyngeal ducts initially, and they gradually expand and develop, often through the thymus area (thymic cysts in children may be located in the neck) [8, 9]. Acquired thymic cysts are usually caused by inflammation, some immune diseases (e.g., AIDS, lupus erythematosus), or after tumor radiotherapy, or thoracotomy. They are often multilocular in shape [10, 11]. In the present study, all the cysts were located in the thymus area. One case was pathologically diagnosed as a multilocular thymic cyst after surgery, with no underlying disease being found. The remaining cysts were congenital thymic cysts.

Most of the existing literature reports that thymic cysts are typically triangular or round in shape, with smooth edges, rare lobes, and smooth junctions with the pleura. Some thymic cysts can display changes in shape between different scan phases [3, 5, 12, 13]. Most cases in the present study were triangle/chestnut-shaped (50%) or round (29%), and the majority of the cysts had smooth edges. One case had rough edges, and the pathological results indicated type-A thymoma. One case showed lobular changes, which is consistent with other reports in the literature. The thymic cysts in the present study were all soft in texture.

In thymic cysts, the sides of the anterior mediastinal thymus area are the parietal pleura, and the insides of the cysts are comprised of loose connective tissue filled mainly with fat cells. The morphology of the pleura changes with the pulling of the bilateral pleura during breathing. The morphological changes of some cysts reported in the literature and in the present study between several instances of examination are generally considered to reflect the difference in the inspiratory volume of the lungs during each examination, with the changes in the shape of the cyst caused by the different positions of the bilateral mediastinal pleura (due to pulling and stretching). A small number of cysts with morphological changes were observed in the present study (3/24), which might be due to the end-inspiratory breath-hold technique adopted for the chest CT scan in the present study. The inspiratory volume in each phase was relatively fixed; thus, any changes in the position of the pleura might not be obvious. Because the texture of these cysts is relatively soft, the contact surface of the cyst/pleura often manifests the “edge straight sign,” and the morphology of the pleura generally does not change. It is mentioned in the literature that some large thymic cysts can exert pressure on the pleura, causing them to become swollen with a clear edge [8, 12]. The cysts in the present study were all small in size—the largest measured axial length was 32.1 mm—and no obvious pleural bulges were detected.

The CT value of thymic cysts is often an atypical liquid density, which is why these cysts are often misdiagnosed as thymoma or other types of solid mass. It is reported in the literature that approximately 38%–83% of thymic cysts have a CT value of  > 20 HU and the highest reported CT value is 97 HU. A noncontrast enhanced-scan CT value of  > 20 HU has been identified as the main reason for the misdiagnosis of thymic cysts before surgery [5, 6, 14]. In the present study, there were 19/24 cases (79%) with noncontrast enhanced-scan CT values > 20 HU, and the highest CT value was 81 HU; these figures are close to those reported in the literature. A relatively small proportion of the cysts in the present study showed typical liquid density (21%), which was also the main reason for the high misdiagnosis rate based on the preoperative images. It was also found that 15 patients (15/24, 63%) showed a CT value reduction in the enhanced scan, with a maximum CT value reduction of 40 HU. Five cases displayed a significant increase in the CT value. Thus, there were only four cases in which the CT value did not change significantly. These findings differ from much of the existing literature, with other studies typically finding no obvious enhancement or only slightly increased CT values in the enhanced scan [5, 8, 12, 13].

We failed to find relevant literature discussing cases of decreased CT value of the thymic cysts in the enhanced scan compared with the noncontrast enhanced scan. We speculate that the observed reductions in CT value in the enhanced scan in the present study might be ultimately due to the soft texture of the thymic cysts. The pulmonary inspiratory volume is different during each different phase of the scan, which causes the pleura of the bilateral anterior mediastinum to stretch and pull the thymic area to different degrees, which in turn changes the morphology and density of the contents inside the cyst. When the inhalation is adequate, the thorax is fully expanded, and the tension of the cyst increases, resulting in decreased density (and vice versa). In the present study, there were two patients with enhanced scans and one of them had obviously insufficient inspiration (Figure 1(e), observed by the tracheal morphology), the CT value of cyst was significantly higher than that of inspiratory state; this is, consistent with the above speculation. Furthermore, four cases with an increased CT value in the enhanced scan may be attributable to “false enhancement” signs caused by the change in the morphology of the cyst. Changes in the cyst morphology can be detected by comparison between the CT scans, but subtle changes in the cyst morphology are difficult for imaging doctors to identify. Changes in the density of the ROI area in the cyst can be distinguished by CT to a higher degree of sensitivity than changes in the cyst morphology.

Chest magnetic resonance (MR) examination is being used more frequently in clinical practice due to its advantage of involving no electromagnetic radiation [15, 16]. However, the thymus area is close to the main blood vessels of the heart and is also affected by respiratory artifacts. The time taken for a single examination usually exceeds 40 minutes, which places high demands on the patient in terms of breath-holding duration and tolerability. Therefore, it is not clinically used as the preferred method for the examination of masses in the thymic area; chest CT scanning is still the recommended technique for examining this area [5]. With the adjustment of the MR scanning sequence, it will play a more important role in the diagnosis of masses in the thymic area.

Due to the issue of atypical density, the main concern in the differential diagnosis of thymic cysts is thymoma. Larger thymomas can more easily be distinguished from thymic cysts due to their uneven internal density, progression, and invasion of the surrounding structures, combined with more obvious enhancement. However, the density of smaller thymomas is relatively uniform, and there exist significant image overlaps between thymic cysts and some mildly enhanced tumors. This is also the reason for most cases of misdiagnosis of thymic cysts as thymoma [6, 13, 17].

The present study's findings identified reduced CT values in the imaging features of some thymic cysts during the enhanced scan, which were not seen in other thymic tumors or lesions. This imaging sign might become an important basis for the diagnosis of thymoma to help improve the identification rate of thymic cysts and reduce unnecessary surgical trauma. The sample size of this study was small, and it was a single-center study; and the cysts in the samples were small, so it was easy to be affected by the partial volume averaging when the conventional 5 mm slice thickness was selected, resulting in the measurement deviation, which was also a shortcoming of this study.

## Figures and Tables

**Table 1:** CT findings in 24 cases with thymic cysts.

CT finding	*N* (*n* = 24)
Location	
Centered	17 (71%)
To the left	3 (13%)
To the right	4 (16%)

Morphology	
Triangle/chestnut shape	12 (50%)
Round/round-like	7 (29%)
Irregular/flat	5 (21%)
With changes in shape at the triple-phase scan	3 (13%)
Without changes in shape at the triple-phase scan	21 (87%)

Edge	
Smooth	20 (83%)
Rough or lobulated	3 (13%)
Cyst wall calcification	1 (4%)

Whether or not close to the pleura	
Not close	10 (42%)
Being close unilaterally	10 (42%)
Being close bilaterally	4 (16%)

Whether or not pull the pleura (knife-cutting sign)	Without pulling the pleura in14 cases

The value in the plain CT of the ROI above 20HU	19 (79%)

Comparison of the value in the plain CT of the ROI with that in the enhanced CT scan	
With the enhanced CT value lower than that in the plain scan	15 (63%)
No significant changes in the CT value between enhanced scan and the plain scan	4 (16%)
With the enhanced CT value higher than that in the plain scan	5 (21%)

## Data Availability

The data used to support the findings of this study are available from the corresponding author upon request.
